# Neuroimaging lesion assessment by pseudo-subtraction of overlaid semi-transparent volumes: A technical description and feasibility series

**DOI:** 10.1177/1971400920975730

**Published:** 2020-12-02

**Authors:** Anna Falk Delgado, Alberto Falk Delgado

**Affiliations:** 1 Department of Clinical Neuroscience, Karolinska Institutet, Sweden; 2 Department of Neuroradiology, Karolinska University Hospital, Sweden; 3 Department of Surgical Sciences, Uppsala University, Sweden

**Keywords:** Co-registration, CT, follow-up, MRI, subtraction

## Abstract

Assessing and reporting clinical images constitutes the mainstay of clinical neuroradiology. Continually increasing numbers of neuroradiology referrals and follow-up examinations call for reproducible, accurate, and rapid workflows. Readily available and easy to use, advanced workstation tools such as co-registration of volume series can be used to overlay volume series from two different time points as semi-transparent images, with an inverse color scale. By overlaying semi-transparent inverse color maps, stationary findings will be shaded out in grey, whereas progressing or regressing lesions will be highlighted as white or black in the resulting pseudo-subtraction map. Pseudosubtraction in longitudinal neuroradiology imaging might enhance workflow and imaging assessment.

## Introduction

Subtraction of magnetic resonance (MR) images has been described in multiple sclerosis to enhance detection of contrast enhancing lesions, estimate lesion volume change,^1^ or assess new lesions in longitudinal follow-up.^2^ Subtraction of images requires dedicated software not readily available at all imaging centers. Here, we present a simple technique that instead of subtraction utilizes overlaid semi-transparent greyscale volume images from two different time points, namely, routine follow-up. The term pseudo-subtraction implies the resulting image will not be a true subtracted image but a result of two overlaid semi-transparent (50%) volumes with opposite grey scales. By applying inverse grey-scale stationary findings will be shaded out in grey whereas progressing or regressing changes will be highlighted in black and white, respectively. Using this approach, a simple co-registration software that is available in most radiology departments can be used in clinical practice to enhance workflow and imaging assessment.

## Neuroradiology pseudosubtraction

**Figure 1.**
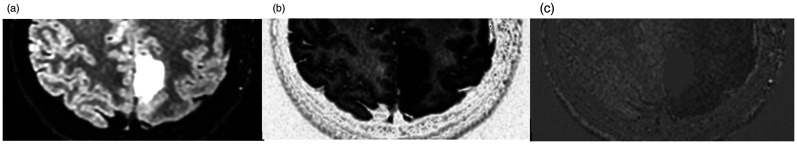
(a) Baseline investigation for an oligodendroglioma World Health Organization grade II on 3D-T2FLAIR (greyscale), with (b) follow-up images on 3D-T2FLAIR (inverse grey) and (c) pseudo-subtraction of the two volumes in GE AW server. As depicted in (c), the findings between examinations (a) and (b) are stationary, which is represented by the grey area given the inverse relationship between the overlaid semi-transparent three-dimensional volumes. GE AW: General electric healthcare advanced workstation.

**Figure 2.**
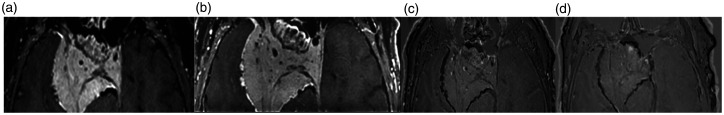
(a) Baseline investigation for skull-base meningioma on 3D-T1Gd with (b) follow-up images on 3D-T1Gd and (c) pseudo-subtraction of the two volumes in GE AW server and (d) Philips IntelliSpace Portal. A clearly visible dark rim around the central grey core represents the size progression of the tumor. Surrounding stationary findings are grey due to the inverse relationship between the overlaid three-dimensional volumes (volume (a) greyscale, volume (b) inverse grey). GE AW: General electric healthcare advanced workstation.

**Figure 3.**
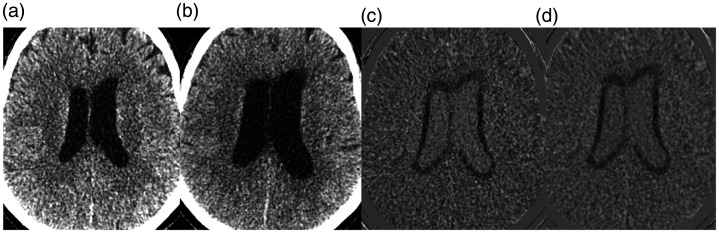
a, Baseline investigation for ventricular size estimation on nonenhanced computed tomography (CT) with (b) follow-up images on nonenhanced CT and (c) pseudosubtraction of the two volumes in GE AW server and (d) Philips IntelliSpace Portal. As shown in (c) to (d), the dark rim around the ventricles represents their size progression. Surrounding stationary findings are grey due to the inverse relationship between the overlaid three-dimensional volumes (volume (a) inverse grey, volume (b) greyscale). GE AW: General electric healthcare advanced workstation.

**Figure 4.**
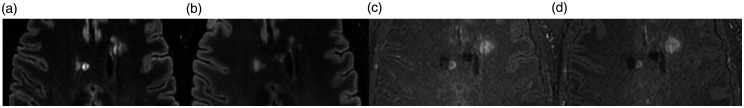
(a) Baseline investigation for multiple sclerosis on 3D-T2FLAIR with (b) follow-up images on 3D-T2FLAIR and (c) pseudosubtraction of the two volumes in GE AW server and (d) Philips IntelliSpace Portal. The dark lesions in (c) and (d) represent a new or increasing size of a previous lesion. The white lesions represent regression of previous lesions. Surrounding stationary findings are grey due to the inverse relationship between the overlaid three-dimensional volumes (volume (a) greyscale, volume (b) inverse grey). GE AW: General electric healthcare advanced workstation.

Both volume series from computed tomography and MR imaging, with or without contrast, can be used to create a pseudosubtraction map. For this technical note, two software packages featuring co-registration with overlaid volumes were used: GE Healthcare AW server 3.2 (Chicago, Illinois, US) and Philips Healthcare IntelliSpace Portal version 9.0 (Amsterdam, the Netherlands). When co-registering overlaid volumes from two different time points, one of the volumes is set to “standard grey scale” (Figure 1(a)) and the other volume to “inverse grey scale” (Figure 1(b)). The transparency of the overlaid volume should be set at 50%. This will make stationary findings appear grey based on overlaid black versus white regions with 50% transparency. Progressing and regressing lesions will be highlighted in either white or black, depending on the type of images, use of contrast agent, anatomic region, and color scale settings. Unchanged volumes will result in all grey (Figure 1(c)). For standardization purposes, we recommend that progressive changes are highlighted in black and regression is highlighted in white. Figures 2–4(a) to (d) show the application of this workflow in three different diagnoses common in serial imaging. For this technical note, ethical approval was obtained and informed consent was waived.

## Discussion

This work presents an easy-to-use application of co-registration software that can be used in longitudinal assessment of neuroradiology images. Applying an overlaid volume assessment simplifies reading follow-up studies by only highlighting changes. Without this new tool, analyzing follow-up imaging can be time consuming, for example, due to subtle changes in lesion volume not readily accessible in standard axial stacks. The method described is particularly suitable for complex and multiple lesions such as skull-base meningiomas, the ventricular system, or multiple sclerosis plaque assessment. For multiple lesions, comparing two sets of images can be demanding, with the need to continually assess the two series at every lesion location. In an overlaid volume, only one volume series will be assessed with the potential to enhance workflow and increase accuracy. Potential benefits from applying the pseudo-subtraction technique can be more fast and robust assessment of complex or multiple lesions. It also has the potential to be automatized with computerized reading. 
